# Early Olfactory Environment Influences Social Behaviour in Adult *Octodon degus*

**DOI:** 10.1371/journal.pone.0118018

**Published:** 2015-02-11

**Authors:** Natalia Márquez, Jaime Martínez-Harms, Rodrigo A. Vásquez, Jorge Mpodozis

**Affiliations:** 1 Instituto de Ecología y Biodiversidad (IEB), Departamento de Ciencias Ecológicas, Facultad de Ciencias, Universidad de Chile, Santiago, Chile; 2 Departamento de Biología, Facultad de Ciencias, Universidad de Chile, Santiago, Chile; University of Pisa, ITALY

## Abstract

We evaluated the extent to which manipulation of early olfactory environment can influence social behaviours in the South American Hystricognath rodent *Octodon degus*. The early olfactory environment of newborn degus was manipulated by scenting all litter members with *eucalyptol* during the first month of life. The social behaviour of sexually mature animals (5–7 months old) towards conspecifics was then assessed using a y-maze to compare the response of control (naïve) and treated animals to two different olfactory configurations (experiment 1): (i) a non-familiarized conspecific impregnated with *eucalyptol* (*eucalyptol* arm) presented against (ii) a non-familiarized unscented conspecific (control arm). In addition, in dyadic encounters, we assessed the behaviour of control and *eucalyptol* treated animals towards a non-familiarized conspecific scented with *eucalyptol* (experiment 2). We found that control subjects explored and spent significantly less time in the *eucalyptol* arm, indicating neophobic behaviours towards the artificially scented conspecific. Treated subjects explored and spent similar time in both arms of the maze, showing the same interest for both olfactory stimuli presented. During dyadic encounters in experiment 2, an interaction effect between early experience and sex was observed. Control males escaped and avoided their scented partner more frequently than *eucalyptol* treated male subjects and than females. Both groups did not differ in the exploration of their scented partners, suggesting that avoidance within agonistic context does not relate to neophobic behaviours. Our results suggest that the exposure to *eucalyptol* during early ontogeny decreases evasive behaviours within an agonistic context as a result of olfactory learning. Altogether, these results indicate that olfactory cues learned in early ontogeny can influence olfactory-guided behaviours in adult degus.

## Introduction

Experience during early ontogeny determines a wide range of behaviours observed in adult animals. In birds, for example, social attachment towards the mother or brood members occurs within a critical period during early ontogeny. Classical experiments performed by Lorenz (1937) and Gottlieb (1961) revealed that such attachment can develop towards any object moving away (e.g. duck decoy, human, among others) presented within this critical period [1, 2]. In ferrets, early olfactory and feeding experiences seem to determine feeding preference of adults [3]. In the same sense, olfactory preferences in rats are formed during early ontogeny in a critical period [4]. The social context under which they are exposed to a given stimulus seems to be determinant for future development of preferences and learning [5, 6]. For instance, in rats and guinea pigs, it is possible to induce adult olfactory preferences towards an artificial odorant if this odorant is presented in the mother’s nipples during the suckling period [7–9]. However, the sole presence of an artificial odorant in the cage during the critical period does not result in the development of olfactory preference by adults [10]. In line with these studies, it has been shown that neophobia towards an artificial odorant, normally observed in adult rats [11], is absent in animals that experienced the odorant on the nipples of their mother during the suckling period [10].

Even though olfaction has been shown to be fundamental in conspecific and kin discrimination in many rodent species [12–14], the role of social context and early olfactory learning on the development of olfactory preferences towards conspecifics has not yet been evaluated. Studies on kin discrimination have mostly focussed on the mechanisms underlying behavioural bias towards genetically related conspecifics (see [12, 15–20]). From those studies, different mechanisms have been proposed by which individuals can discriminate between kin and non-kin (see [12, 13, 15, 19, 21–23]). Currently, the most accepted mechanisms are: (1) recognition by prior association, which considers that the behavioural bias is determined by familiarity (i.e. common living), and (2) recognition by phenotype matching, where individuals learn cues that are shared by family members, treating individuals with those cues as kin [15, 22]. It has been proposed that learning of distinctive signals is involved in both of these mechanisms, whether related with kinship or not, and that those mechanisms could act together for kin recognition [13, 22].

In the present study we used the social rodent *Octodon degus* to investigate the effect of early olfactory experience on social behaviour of adults. Degus are diurnal and highly social semifossorial caviomorph rodents, endemic to central Chile [24]. Several characteristics make degus a suitable model to study social behaviours, including communal nesting [25–27], group foraging [28, 29] and their behavioural bias towards their kin mainly based on direct familiarization [14]. Taking advantage of the highly social characteristic of degus, we studied whether early olfactory experience can influence social interactions between adult animals sharing olfactory signals experienced during early ontogeny. We manipulated the olfactory environment of newborn *O. degus* by impregnating litters with *eucalyptol* during the first month of life, in order to assess if early experience to this artificial odorant can result in behavioural changes in adult degus. Potential olfactory preferences and behavioural biases towards a non-familiarized conspecific impregnated with *eucalyptol* were then tested on 5–7 months old individuals. Here we show that olfactory cues experienced during early ontogeny have a long lasting effect and can determine social behaviours of adult degus.

## Materials and Methods

### Subjects

Males and females *O. degus* born and reared in our colony were used. Dams and litter were housed in metal cages (50 x 40 x 35 cm) with wood shaving, under natural photoperiod in two different air-conditioned rooms at the Faculty of Sciences, University of Chile. Animals were fed with alfalfa and rabbit pellets, and water was provided *ad libitum*. Both maintenance and experimental procedures were approved by the ethics committee of the Faculty of Sciences of the University of Chile, and followed Chilean regulations.

### Rearing conditioning

From postnatal (PN) day one, the mother was rubbed on the anterior and ventral area with cotton balls impregnated with *eucalyptol* (C80601, Aldrich), 3.3 μM between 10–15 times. From PN2 to PN30, pups and dams were daily rubbed in the ventral and dorsal area with the artificial odour. After this exposure period, dams and siblings remained together in their home cages and only experienced *eucalyptol* once again in the experimental session.

### *Y-maze* (experiment 1)

**Fig 1:**
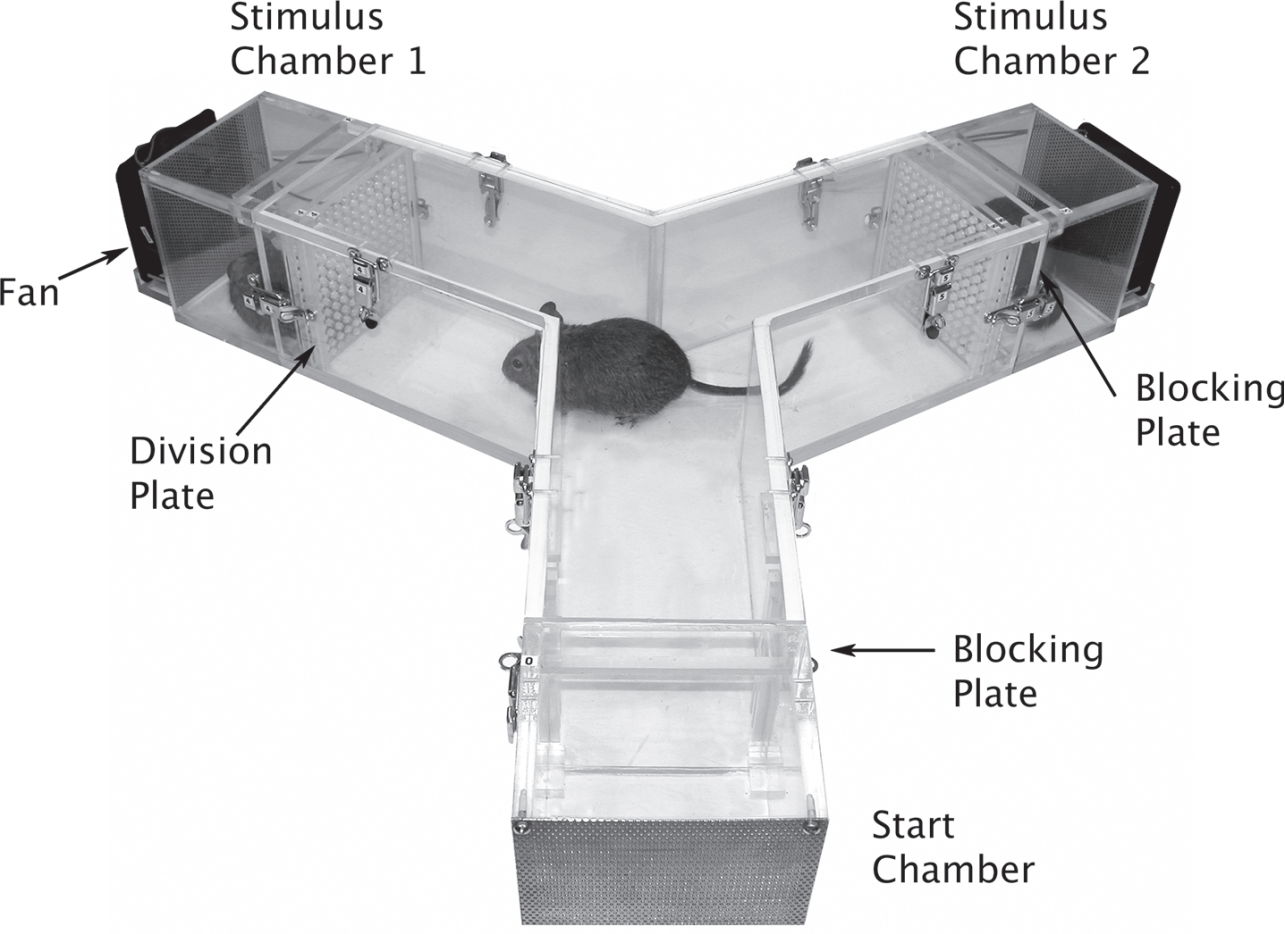
The *y-maze*. The system was designed to allow a focal *O. degus* to explore freely the three arms of the maze. Stimulus subjects were placed in chambers attached to the extremes opposite to the start chamber. A division plate prevented direct contact between focal and stimuli subjects, but allowed the airflow produced by a fan behind each stimulus chamber to circulate through the labyrinth.

Treated degus (exposed to *eucalyptol* during the suckling period) as well as control animals (naïve to *eucalyptol*) were tested in a *y-maze* (Fig. 1). This experiment allowed us to present two olfactory stimuli simultaneously, and therefore assess potential behavioural differences within and between groups towards both olfactory configurations. In each stimulus chamber a non-familiarized conspecific (i.e. reared apart) was placed, but only one of them was impregnated with *eucalyptol*. The maze consisted of three Plexiglas arms (50 cm long x 15 cm width x 15 cm high forming angles of 120°). A perforated Plexiglas plate (division plate) prevented direct contact between focal and stimulus subjects, but allowed the airflow produced by a fan behind each stimulus chamber to circulate through the *y-maze*. During an acclimatization period a Plexiglas plate (blocking plate) avoided the movement of the focal subject, and also prevented volatile odours from the stimulus chambers to reach the start chamber. Once the experiment began, the blocking plates were removed, and the fans next to the stimulus chambers were switched on. Stimuli and tested animals were from the same sex; a total of 24 animals were tested only once, 6 females and 6 males per group. Treated and control individuals were reared in colonies housed in different rooms to ensure that control animals had no experience with the odorant prior to the test; animals were carried in individual plastic cages from the housing rooms to the experimental room. The experiment consisted in 1 min acclimatization period and 3 min test. To avoid scent contamination between animals, we used different sets of gloves to manipulate animals in the maze. Additionally, the *y-maze* was cleaned with 95° ethanol to eliminate odour traces after each trial. Position of the stimulus was randomized in order to control for maze side preference. All experiments were video recorded (colour CCTV camera connected to a Sony video recorder) from above the *y-maze* system. An observer blind to the treatment and subject sex analysed the recordings using the JWatcher 1.0 software (Dan Blumstein, University of California, Los Angeles, U.S.A). The behaviours evaluated were exploration time and total time (still and exploring) spent by the animals in each arm.

### Pair encounters (experiment 2)

In order to assess behavioural bias during pair encounters we compared exploratory, social and aggressive behaviours (see below) of animals that differed in their previous experience with *eucalyptol*. Animals were always tested with a non-familiarized conspecific (i.e. not genetically related nor reared together) impregnated with the artificial odorant in an experimental arena during 10 min. From a total of 28 pairs of animals of the same sex, 7 male and 7 female pairs were assigned to each treatment. Experiments were carried out as described by Villavicencio et al. (2009), in two 80 x 80 x 50 cm metallic arenas that could be divided by placing a division plate. The floor consisted of a removable white-painted metal plate that was cleaned with detergent between tests to remove any trace of scent that could have been left by previous pairs. The focal subject was marked with non-toxic painting, which allowed an observer to distinguish between the two animals when analysing the experimental video recordings; previous studies have shown no change in locomotion, vigilance [30] or exploratory behaviour [14] due to marking. The stimulus animal was impregnated with *eucalyptol* as described for experiment 1. After a 10 min acclimatization period, the division plate was removed and behavioural quantification began when the focal subject started to explore the arena. All experiments were video recorded, and afterward analysed with the JWatcher software. Three behavioural categories were defined, based on behavioural descriptions of intraspecific interactions in degus [24, 31–33] and mice [34]: (1) social exploratory behaviour consisted in exploratory approaches to the mouth, head, flanks and/or anogenital area of its partner; (2) cohesive behaviour, was considered when degus were in contact either side by side, on right angle to each other, grooming, or huddling one over the other, and (3) agonistic encounters of two kinds, (i) evasive, whenever a focal subject by turning aside or running away avoided the partner in hostile contexts, and (ii) aggressive, if the animal performed tail wagging, hindleg kicking, foreleg pushing, chasing or fighting with the partner. Additionally, we chose a non-social behaviour, exploration of the arena, to address if the presence of the artificial odorant in the experimental arena can affect other kind of behaviours.

### Statistics

**Experiment 1**

One animal was not considered in the statistical analysis because remained still during the entire experiment. To evaluate the effects of early experience, comparisons between groups were analysed with one-way ANOVA or Kruskal-Wallis test depending on whether the data met parametric requirements or not. Intra-group comparisons were analysed using paired *t*-test or Wilcoxon Signed-Rank test depending on normality of the dependent variables [35]. For all the analysis we used the software R (R Foundation for Statistical Computing, Vienna, Austria 2009).

**Experiment 2**

Two animals were not considered in the statistical analysis because they remained still during the entire experiment. Given that the data from experiment 2 did not accomplish parametric requirements, the Scheirer-Ray-Hare test, extension of the Kruskal-Wallis test for non-parametric data, was employed to analyse the results [35].

## Results

The aim of the experiments was to test whether the experience with an artificial odorant during early ontogeny influenced social and olfactory guided behaviours in adult animals.

### *Y-maze* (experiment 1)

**Fig 2:**
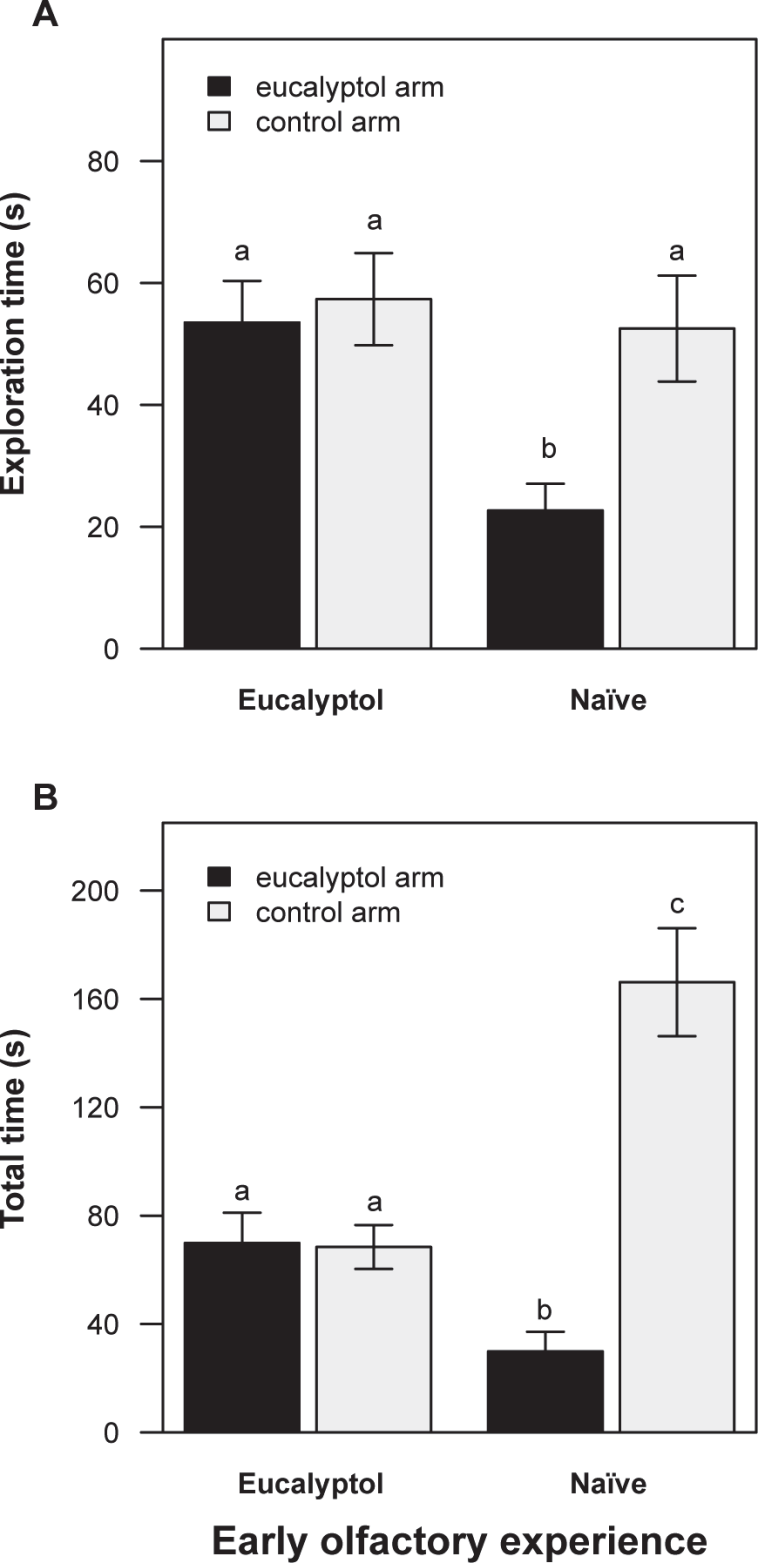
Effects of early olfactory experience on *O. degus’* behaviours in the *y-maze*. (A) Exploration time (mean ± SE) of the *y-maze* arms by animals with different olfactory experience during early ontogeny (*eucalyptol* or naïve). Naïve subjects (controls) showed neophobia towards the artificial olfactory configuration (a conspecific impregnated with *eucalyptol*), exploring significantly less the *eucalyptol* arm (in black), subjects reared with *eucalyptol* (treated) explored similarly both arms. (B) Total time (mean ± SE) spent by both groups in each arm. Control subjects remain significantly more time in the control arm (in grey) and spent significantly less time in the *eucalyptol* arm than *eucalyptol* experienced subjects. Different letters represent statistically significant differences between the groups in each arm (*p* < 0.05, see text for exact statistical and *p* values).

Treated and control animals (naïve to *eucalyptol*) were tested in a *y-maze* (Fig. 1). First we performed comparisons within groups to address potential differences in the exploration time towards the two olfactory stimuli presented. We found that the control subjects spent significantly less time exploring the arm of the *y-maze* containing the artificially scented conspecific (paired *t*-test: *t* = 3.65, *p* = 0.004; Fig. 2A). For *eucalyptol* treated animals, on the other hand, no significant differences were found in the time spent exploring the two arms (paired *t*-test: *t* = 0.41, *p* = 0.69; Fig. 2A). Additionally, we performed comparison between groups to compare the exploration time for each arm between treated and control animals. Fig. 2A shows that control degus explored the *eucalyptol* arm significantly less than treated animals (ANOVA: *F*_(1, 21)_ = 7.08, *p* = 0.002), yet both groups explored the control arm similarly (ANOVA: *F*_(1, 21)_ = 0.09, *p* = 0.77).

The comparison of the total time (i.e. time exploring and time still) spent in each arm (Fig. 2B) reveals that control subjects spent significantly more time in the control arm than in the *eucalyptol* arm (paired *t*-test: *t* = 3.78, *p* = 0.004). Compared to treated subjects, control degus spent significantly less time in the *eucalyptol* arm (Kruskal-Wallis test: *H* = 5.48, *p* = 0.02) and significantly more time in the control arm (ANOVA: *F*_(1, 21)_ = 10.66, *p* = 0.004). In contrast, treated degus showed no significant differences in the time spent exploring both arms of the *y-maze* (paired *t*-test: *t* = -0.076, *p* = 0.94). Altogether, our results indicate that control degus avoided the *eucalyptol* arm, suggesting that they presented neophobia towards the artificial olfactory configuration. In contrast, no signs of neophobia were observed in animals that experienced e*ucalyptol* during the suckling period.

### Paired encounters (experiment 2)

**Fig 3:**
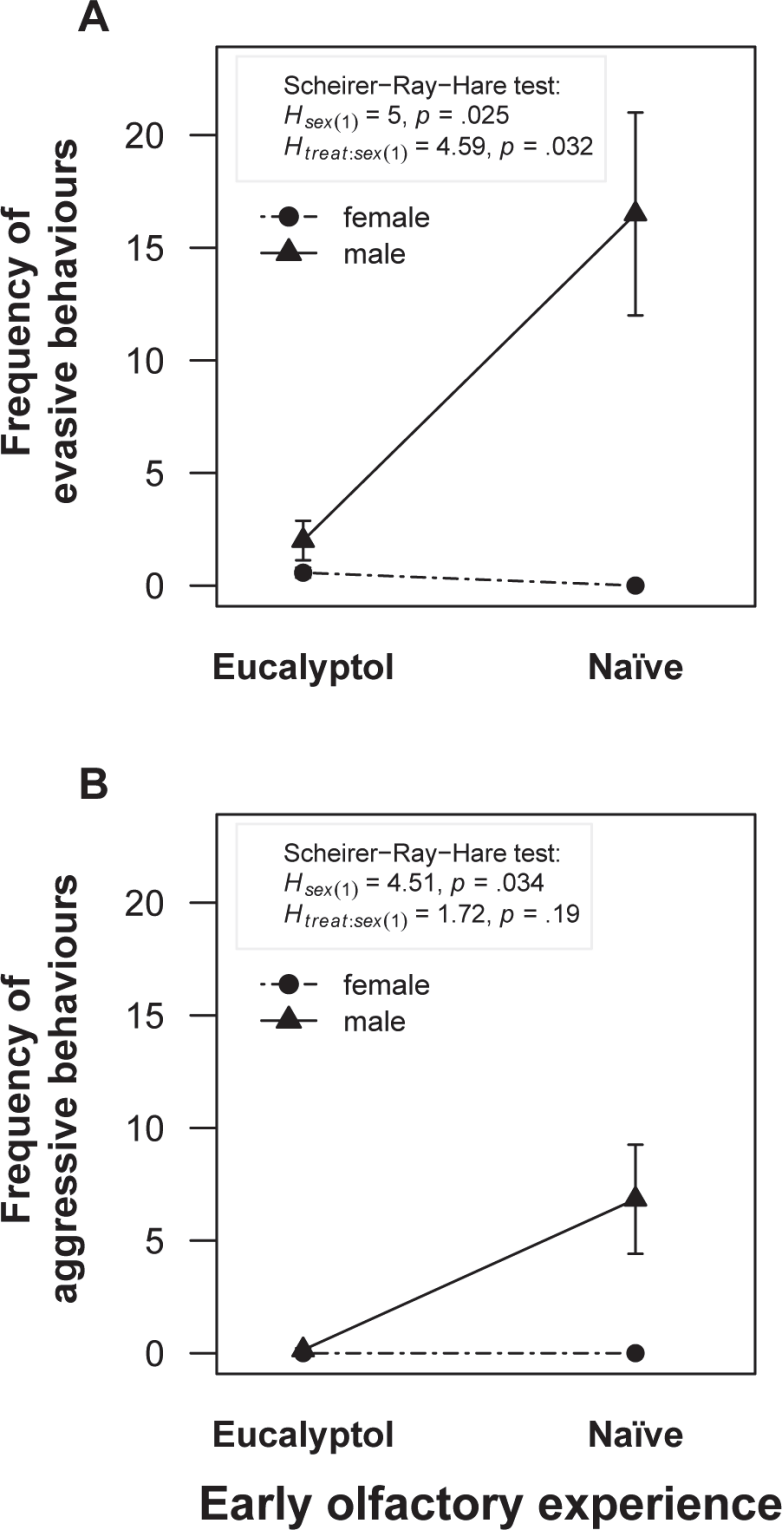
Agonistic display during dyadic encounters between animals of the same sex. (A) Frequency of evasive behaviour (mean ± SE) depended on the sex and previous experience with the artificial odorant. Naïve males performed this kind of behaviours significantly more. (B) Aggressive behaviours (mean ± SE) were more frequent in males than in females. *H* and *p*-values from Scheirer-Ray-Hare test are shown in each figure.

Control (naïve) and treated degus were compared with respect to their behaviour towards a non-familiarized conspecific impregnated with *eucalyptol* during dyadic encounters. We observed that not all quantified behavioural categories (see methods) were affected by early olfactory experience in the same way (see Table 1). Agonistic encounters of either type (i.e. evasive or aggressive) were not frequent in females. The effect of early experience on evasive behaviour (escape and run away) depended on the sex of the subject, as shown by the interaction effect between early experience and sex (Scheirer-Ray-Hare test; sex: *H* = 4.996, d.f. = 1, *p* = 0.025; interaction: *H* = 4.59, d.f. = 1, *p* = 0.032; Fig. 3A). Control males escaped and avoided their scented partner more frequently than both female groups, and than treated males. Control males tended to perform aggressive behaviours more frequently than all other groups, but no significant effect of the interaction between sex and olfactory experience (interaction: *H* = 1.72, d.f. = 1, *p* = 0.19; Fig. 3B) was found. We found a significant sex effect on aggressive behaviours, with males performing aggressive behaviours significantly more frequently than females (sex: *H* = 4.51, d.f. = 1, *p* = 0.034; Fig. 3B).

**Table 1:** Effects of rearing condition and sex on different behavioural categories (mean ± SE) during dyadic encounters in *O. degus*.

		Rearing condition			
		Control (naïve)		Treated (*Eucalyptol*)	
Behavioural categories	Behaviour	Females n = 6	Males n = 6	Females n = 7	Males n = 7
Agonistic encounters (frequency)	Evasive	0 ± 0	16.5 ± 4.5	0.57 ± 0.22	2 ± 0.78
	Aggressive	0 ± 0	6.83 ± 2.4	0 ± 0	0.14 ± 0.07
Social exploration (s)	Anterior	13.4 ± 1.8	33.3 ± 5.3	6 ± 1.4	24.7 ± 5.3
	Anogenital	9 ±3.2	16.7 ± 3.9	1.4 ± 0.5	12.8 ± 3.3
	Flank	16.7 ± 3.7	17.9 ± 3.9	6.7 ± 1	22.3 ± 2
Social contact (s)	Cohesive	92 ±33.3	86.7 ± 38.4	68.9 ± 25.8	152 ± 49.3
Others (s)	Non-social exploration	113 ±13.2	69.6 ±12.4	43.2 ± 14.4	77.2 ± 19.6

**Fig 4:**
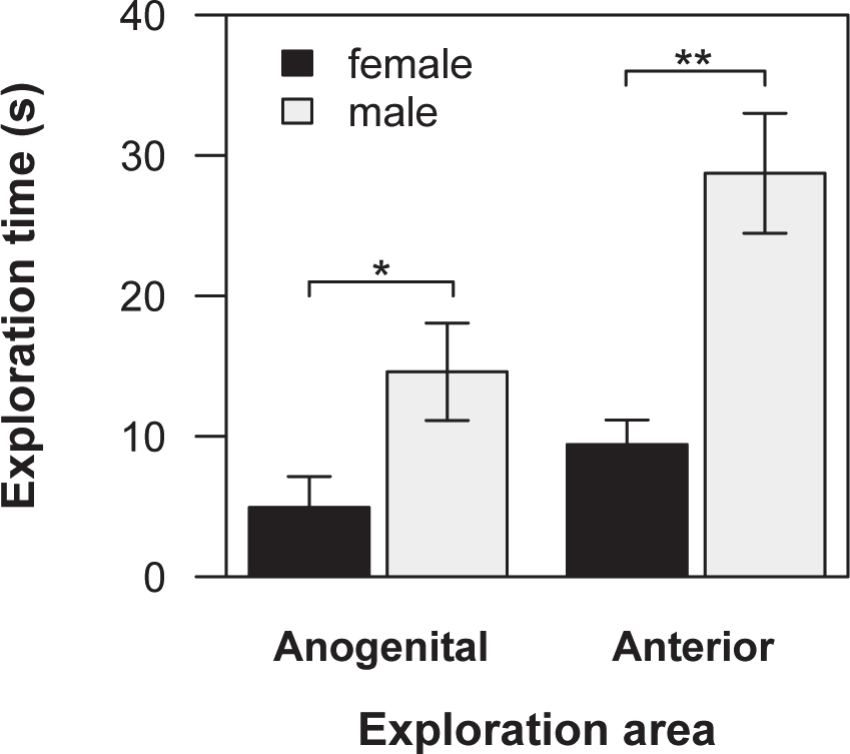
Exploration time (mean ± SE) during dyadic interactions between individuals of the same sex. Males (in grey) explored significantly more the anogenital and anterior area of their partners (a non-familiarized conspecific impregnated with *eucalyptol*) than females (in black). Asterisks represent significant differences between males and females, Scheirer-Ray-Hare test (*P < 0.05 and **P < 0.01).

An additional behavioural category quantified in this experiment was social exploration. We found no effect of the rearing condition on exploration in any of the three body regions quantified (Scheirer-Ray-Hare test for rearing condition, anterior region: *H* = 1.1, d.f. = 1, *p* = 0.3; flank *H* = 0.4, d.f. = 1, *p* = 0.5; anogenital region: *H* = 1.34, d.f. = 1, *p* = 0.24). We found a sex differences in the exploration of two body regions during dyadic encounters. Males explored a non-familiarized conspecific impregnated with *eucalyptol* significantly longer than females (see Table 1, Fig. 4) in the anogenital (Scheirer-Ray-Hare test, sex: *H* = 5.19, *p* = 0.023) and anterior region, which included head and nose (Scheirer-Ray-Hare test, sex: *H* = 7.25, d.f. = 1, *p* = 0.007). Non-social behaviours, i.e., exploration of the experimental arena, were not affected by the presence of *eucalyptol* (Scheirer-Ray-Hare test, trat: *H* = 1.94, d.f. = 1, *p* = 0.16, sex: *H* = 0.08, d.f. = 1, *p* = 0.78, interaction: *H* = 0.85, d.f. = 1, *p* = 0.35; Table 1).

## Discussion

In the present study we investigated the extent to which early olfactory experience can influence social interactions of adult *O. degus*. We combined techniques used to assess the role of early olfactory experience in olfactory preferences (artificial scenting and *y-maze*) with behavioural experiments used to test social or kin discrimination (pair encounters arena).

Our results revealed behavioural differences between degus developed in a social environment modified by the presence of an artificial odour (*eucalyptol* treated) and degus whose close olfactory environment was not modified (controls). Such differences were observed in a *y-maze* as well as in social interactions during dyadic encounters in an experimental arena. In the *y-maze* (experiment 1), control animals showed neophobic behaviours towards the artificial olfactory configuration, as indicated by the avoidance of the arm containing an individual scented with *eucalyptol*. Additional evidence of neophobia was observed in control animals that remained still significantly longer in the arm containing a conspecific lacking the artificial odorant. On the other hand, degus reared in an artificially scented social environment did not show avoidance for the arm containing a conspecific impregnated with *eucalyptol*; they explored and spent similar time in both arms of the maze. This result suggests that the exposure to an artificial odorant during early ontogeny revokes neophobic behaviours in adult degus towards the artificial odorant. Similar results have been reported in rats tested at post weaning and at PN70 [10]. In contrast, in the experiments presented here, adult degus were tested 5–7 month after they experienced the artificial odour. Taking into account that in natural conditions survival of degus for more than 15 months is 0.7% [36], the age used in our study is representative of an adult degu in the wild. Therefore, our results do not only stress the relevance of early olfactory learning, but also emphasize its long lasting effect.

Our study also revealed an effect of early olfactory experience on dyadic encounters (experiment 2). We observed an interaction effect between sex and early experience on evasive behaviours (avoiding the partner and/or running away). Control males escaped significantly more from the scented partner during agonistic display than all other groups. Although no interaction effect between sex and early experience was found in aggressive behaviours, control males tended to perform more this kind of behaviours than the other tested groups. While no difference in social exploration between control and treated degus was found, males explored their partner significantly more than females. Given that control subjects did not show a reduction of social and non-social exploration, evasive behaviours in this experiment cannot be considered to be of neophobic nature. The lack of differences in exploratory behaviour between control and treated animals suggests that even though the presence of *eucalyptol* can account for a decrease in agonistic behaviours, degus might be able to discriminate the olfactory configuration produced by non-familiar conspecifics even in the presence of a known artificial odorant. Hence, it could be argued that in our experiment the non-familiar conspecific represented a novel stimulus, and that this novelty motivated the active exploration observed towards the scented partner.

Previous studies on degus have reported that males display agonistic behaviours during dyadic encounters. Such behaviours are reduced after repeated encounters with the same conspecific [33]. Field observations, on the other hand, have shown that aggressive behaviours between males increase during the breeding season [37]. In contrast, agonistic behaviours between females from different nests represent only 3% of their social interactions [27]. Our results are in agreement with these previous studies. While agonistic behaviours between females were not frequent in our experiments, control males performed more agonistic behaviours towards a non-familiarized conspecific compared to eucalyptol-treated animals. Our experiments indicate that early olfactory experience can influence agonistic behaviours in adult degus.

Dyadic encounter experiments have been extensively used in the study of kin discrimination [14, 15, 17, 38]. On those studies, discrimination is considered as differences in behavioural response towards kin compared to non-kin [22]. By means of cross-fostering it has been shown that, depending on the species, agonistic behaviours are less frequent towards conspecifics with olfactory signals similar to the nest mates [18], or towards non-relatives reared together [15]. This evidence suggests that learning of distinctive signals, whether associated or not with kinship, participates in kin recognition [20, 22]. In the case of degus, it has been reported that kin discrimination is influenced by familiarity and by phenotypic similarities [14]. Comparable to studies on kin discrimination, our results revealed differences on agonistic behaviours between control males and treated subjects towards a conspecific scented with *eucalyptol*. This suggests that olfactory learning during early ontogeny influences discriminatory behaviour in *O. degus* and reveal that this learning can have a long lasting effect. Until now, kin discrimination studies based on olfaction have successfully demonstrated behavioural bias towards siblings reared apart, indicating that some olfactory features shared between genetically related conspecifics evoke behavioural preferences even in the absence of previous contact between individuals [18]. In the present study, we were able to induce behavioural bias towards non-siblings reared apart by adding an artificial olfactory cue that was present during their early ontogeny in the social context of their suckling period.

A study by Ebensperger et al. (2004) showed that in wild degus nesting communally, groups of two to four females with different degrees of kinship share underground nests [27], each giving birth to between three to eight pups [24]. The same study reported that among above ground female interactions, 97.6% represent co-nesting encounters (all amicable), in contrast to agonistic interactions (less than 3%), occurring exclusively among females from different nests [27]. Considering that early social environment of degus can be conformed by large groups of conspecifics differing in sex and kinship, it can be argue that such early environments provides a rich mixtures of odours from all members of the nest. Previous studies using artificial odorants have shown that olfactory preferences are formed during early ontogeny [4] and that the social context in which the odour occurs is fundamental in their formation [5–10]. Although we did not directly evaluate the role of social context in the formation of behavioural biases, our results suggest that the presence of an odorant within a relevant social context early in ontogeny can influence social behaviour of adult degus. We propose that cooperative or amicable behaviours in degus are mediated by olfaction, and they might be the result of learning of olfactory signals experienced during early ontogeny.

Examples where social attachment or preference occur towards artificial stimuli, clearly illustrate the plastic nature of social attachment and olfactory preferences. This plasticity reveals that what is usually described as normal or typical for a species e.g., the following response of ducklings towards their mother [1, 2], might result from the conservation of an epigenetic history [39]. If we consider epigenesis as a continuous process of structural transformation of an organism as a whole modulated by its interactions with the environment [40, 41], the study of such interactions during early ontogeny results fundamental to understanding behaviour in adults. Previous studies have shown that somatosensorial stimulation (e.g. the grooming of the mother in natural conditions) plays an important role in olfactory plasticity and, consequently, in olfactory learning (see [42]). Given that the development of degus olfactory system might also be affected by somatosensorial stimulation, a great plasticity could be expected in a rich social environment as commonly occurs in natural dens. Under these circumstances, behavioural biases could be established between conspecifics displaying similar olfactory cues, even if no prior social association occurred between them. Considering the highly social characteristics of degus, the role of social context for the formation of behavioural biases needs to be further evaluated. Altogether, our results indicate that early olfactory learning affects social and olfactory guided behaviours in adult degus.
